# Step-Up versus Open Approach in the Treatment of Acute Necrotizing Pancreatitis: A Case-Matched Analysis of Clinical Outcomes and Long-Term Pancreatic Sufficiency

**DOI:** 10.3390/jcm13133766

**Published:** 2024-06-27

**Authors:** Goran Pavlek, Ivan Romic, Domina Kekez, Jurica Zedelj, Tomislav Bubalo, Igor Petrovic, Ognjan Deban, Tomislav Baotic, Ivan Separovic, Iva Martina Strajher, Kristina Bicanic, Ana Ettinger Pavlek, Vanja Silic, Gaja Tolic, Hrvoje Silovski

**Affiliations:** 1 Department of Surgery, University Hospital Centre Zagreb, 10000 Zagreb, Croatia; goranpavlek@gmail.com (G.P.); jurica.zedelj@kbc-zagreb.hr (J.Z.); bubalo.tomislav@gmail.com (T.B.); igor.petrovic33@gmail.com (I.P.);; 2 Department of Oncology, University Hospital Centre Zagreb, 10000 Zagreb, Croatia; domina.kekez@gmail.com; 3 Department of Surgery, University Hospital Dubrava, 10000 Zagreb, Croatia; 4 Department of Anesthesiology and Intensive Care Medicine, University Hospital Centre Zagreb, 10000 Zagreb, Croatia

**Keywords:** pancreatitis, necrosectomy, minimally invasive, surgery

## Abstract

**Background/Objectives**: Acute necrotizing pancreatitis (ANP) with secondary infection of necrotic tissue is associated with a high rate of complications and mortality. The optimal approach is still debatable, but the minimally invasive modality has gained great attention in the last decade as it follows the principle of applying minimal surgical trauma to achieve a satisfying therapeutic objective. We compared clinical outcomes between the step-up approach (SUA) and open necrosectomy (ON) in the treatment of acute necrotizing pancreatitis. **Methods**: A prospective cohort study over the period of 2011–2021 in a university teaching hospital was performed. Results of 99 consecutive patients with ANP who required surgical/radiological intervention were collected. A case match analysis (2:1) was performed, and the final groups comprised 40 patients in the OA group and 20 patients in the SUA group. Demographic, clinicopathologic, and treatment data were reviewed. **Results**: Baseline characteristics and disease severity were comparable between the two groups. The patients from the SUA group had a significantly lower morbidity rate and rate of pancreatic insufficiency. Death occurred in 4 of 20 patients (20%) in the SUA group and in 11 of 40 patients (27.5%) in the ON group (risk ratio with the step-up approach, 0.72; 95% confidence interval, 0.26 to 1.99; *p* = 0.53). **Conclusions**: A minimally invasive step-up approach provides comparable outcomes to open necrosectomy in the treatment of ANP with infected pancreatic necrosis. While mortality and hospital stay were comparable between the groups, morbidity and pancreatic insufficiency were significantly lower in the SUA group. Further studies on a larger number of patients are required to define the place of SUA in the modern treatment of ANP.

## 1. Introduction

Acute necrotizing pancreatitis with a secondary infection of necrotic tissue is associated with a high rate of complications and mortality [1]. Open necrosectomy (ON) was the traditional method to achieve adequate source control and remove the infected necrotic tissue; however, in recent years, less invasive techniques have been developed in order to improve survival and reduce complications. These include percutaneous drainage (transabdominal or retroperitoneal), transgastric drainage, and video-assisted retroperitoneal debridement (VARD) [2,3,4,5]. The authors who designed the PANTER trial introduced the term “step-up approach” to describe the use of these minimally invasive methods, which have the potential to be re-employed toward draining pancreatic necrosis [2,6]. The idea was to initiate necrosectomy using the least invasive methods (and repeat it if necessary) with the aim of avoiding laparotomy and associated morbidity. The PANTER trial and several other studies showed that SUA is a feasible alternative to open necrosectomy [2,3,4,7,8,9,10,11,12]. Furthermore, studies revealed that the minimally invasive step-up approach, as compared to primary open necrosectomy, reduced the rate of major complications and death in these patients. It is postulated that SUA may reduce these rates by minimizing surgical trauma and systemic pro-inflammatory responses in already critically ill patients [5,6].

However, researchers did not thoroughly investigate the efficiency of SUA in preserving viable pancreatic parenchyma and pancreatic exocrine and endocrine functions. Furthermore, the small number of enrolled patients in all these studies, including PANTER, limited the results. Therefore, our single-institution report may be another important step in the clinical and scientific evaluation of ANP treatment. Our study aimed to analyze the short- and long-term outcomes of SUA in our institution and compare them with the outcomes of open necrosectomy. The SUA approach was first performed in our institution in 2011, and 20 patients underwent the procedure over the following decade.

**Figure 1:**
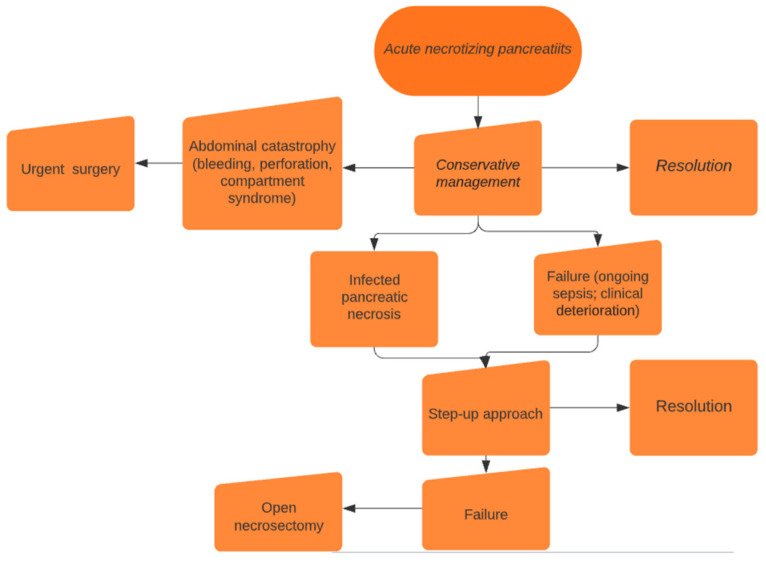
Protocol for acute necrotizing pancreatitis treatment.

**Figure 2:**
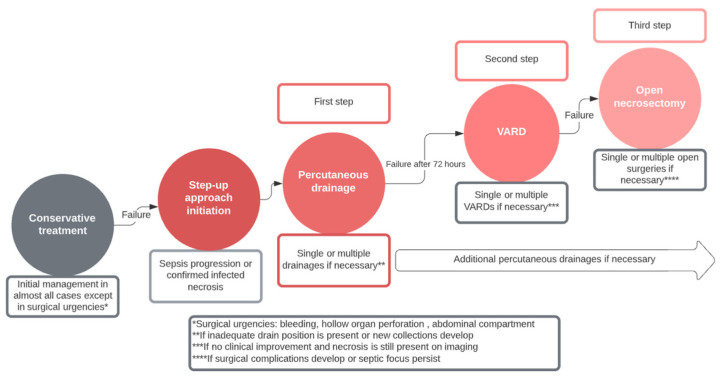
Schematic demonstration of the “step-up approach”.

We followed the strategy described in the PANTER trial and in a study by Sion et al. [7,12]. Suspected infected necrosis should preferably be treated with antibiotics and supportive treatment; in cases of ongoing sepsis, the first step was retroperitoneal percutaneous drainage, which served as a route for minimally invasive retroperitoneal necrosectomy if necessary (Figure 1). In the absence of clinical improvement after 72 h, a second drainage procedure was performed; if there was still clinical improvement after an additional 72 h, the second step, VARD, lavage, and placing of two large-bore drains, was performed. In the case of VARD failure, additional VARD was performed if the patient’s conditions allowed it; otherwise, open necrosectomy was performed as the last resort (Figure 2). If, in any step of this protocol, surgical urgency developed, open surgery was immediately performed. In cases of resolution, no further interventions were required, and conservative therapy was continued. Endoscopic drainage was not available at our institutions; therefore, it was not part of the step-up strategy.

The open necrosectomy included a median or bilateral subcostal laparotomy with the evacuation of intra-abdominal free fluid, careful debridement of pancreatic necrosis, and drainage of the peripancreatic area with two or three large-bore drains, which were used for postoperative lavage. The decision concerning the timing and type of intervention was made by a multidisciplinary team comprised of anesthesiologists, gastroenterologists, and surgeons. This study was designed as a case-matched cohort comparative single-institution study with the goal of analyzing which treatment strategy is optimal in terms of clinical outcomes, safety, and efficiency.

## 2. Materials and Methods

A prospective cohort study in a high-volume university hospital was conducted. Results of 132 consecutive patients aged 18 years or older treated in the Surgical Department during a 10-year period are reported. The analysis included patients with necrotizing pancreatitis and infected pancreatic necrosis undergoing open surgical or minimally invasive necrosectomy between 1 January 2011 and 1 January 2021. The study was approved by the local ethical committee and conducted according to the Declaration of Helsinki.

Patients treated with open surgery were compared to patients who underwent a “step-up approach” in terms of mortality, hospital stay, pancreatic fistula rate, and exocrine/endocrine dysfunction. All the patients had a minimum follow-up of 1 year. The exclusion criteria were postoperative or iatrogenic pancreatitis; abdominal catastrophe as a consequence of AP (bleeding, abdominal compartment, or visceral organ perforation); and patients aged <18. A case-match analysis of 2:1 was performed to avoid comparison and selection bias. The STROBE statement was followed (www.strobe-statement.org, accessed on 14 April 2024.).

Data were extracted from the Hospital Information System and included demographic data (age, sex, body mass index), clinicopathologic data (cause and severity of pancreatitis, ASA score, and comorbidities), laboratory and microbiological data (inflammatory markers, pancreatic enzymes, blood and necrosis cultures), and radiological data (CT findings and CT severity index). In addition, data on the type and number of interventions were recorded. The predefined primary outcome was a comparison between the two groups in terms of mortality (6 months postoperatively), major complications, and the presence of permanent pancreatic insufficiency (at 1-year follow-up). Secondary endpoints were the development of pancreatic fistulas, intensive care unit (ICU) stays, and hospital stays.

Acute necrotizing pancreatitis is defined by necrosis of more than 30% of the gland detected by CT or MRI scan [13]. Infected pancreatic necrosis was defined as a positive culture of pancreatic necrotic tissue obtained surgically or percutaneously by means of fine-needle aspiration or the presence of gas in the peripancreatic fluid collected on radiological imaging. If these criteria were not fulfilled but the patient clinically deteriorated (sepsis progression) despite maximal conservative support, we considered his condition to be suspected infected pancreatic necrosis that required intervention. Major complications are defined as grade > 3b according to the Clavien–Dindo classification of surgical complications [14]. Pancreatic fistula was defined as 30 mL of amylase-rich fluid (3 times more than the upper normal serum amylase value) discharged from drains on or after postoperative day 7. The severity of the fistula was graded according to the International Study Group of Pancreatic Fistula (ISGPF) [15]. Exocrine dysfunction was defined as the need for oral enzyme supplementation to treat diarrhea 6 months after discharge. The endocrine function was evaluated by the glucose tolerance test (GTT), as described by Gasparoto et al. [16].

Patients treated with standard open necrosectomy were compared to those treated by SUA using a 2:1 case-matching (ON: SUA) analysis. Patients were matched for age, gender, and AP severity (according to the Ranson score and CT severity score) to reduce potential biases from confounding variables. The baseline characteristics and primary and secondary endpoints were compared between the treatment groups. Categorical variables are presented as percentages and risk ratios with corresponding 95% confidence intervals, and these were statistically compared with the Chi-square test. Continuous variables are presented as means (±standard deviations) and compared using the *t*-test or Mann–Whitney U test. A *p*-value of <0.05 was considered significant. All data were analyzed with SPSS v27^®^ (IBM, Armonk, NY, USA).

## 3. Results

### 3.1. Study Participants

**Figure 3:**
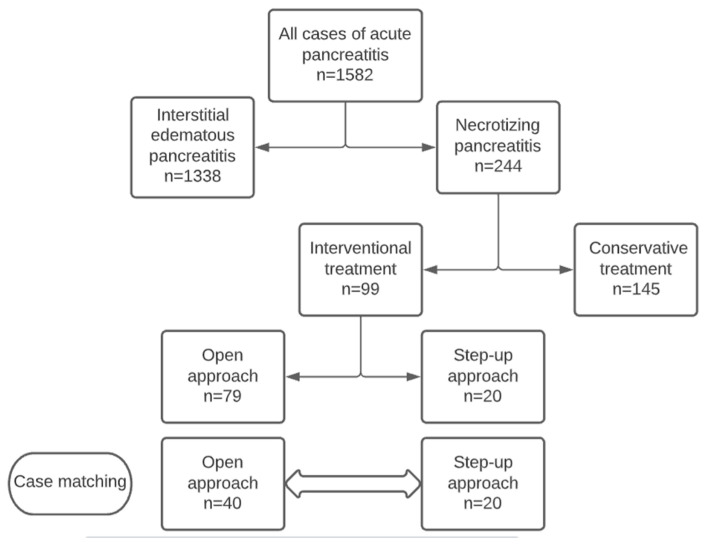
Flowchart of the study.

During the study period, 1582 patients with AP were evaluated and treated at University Hospital Centre Zagreb, and 244 of them were diagnosed with the acute necrotizing type of pancreatitis (Figure 3). Conservative treatment was performed in 145 and invasive intervention in 99 of these patients. The step-up approach was employed in 20 patients, and after a 2:1 case matching for age, gender, and pancreatitis severity score, a comparison between the SUA group (*n* = 20) and ON group (*n* = 40) was performed. 

Patients’ demographic and clinical characteristics are shown in Table 1. There was no significant difference between the groups regarding age, sex, severity scores, and radiologic/laboratory findings. Overall, there were 41 males and 19 females with a mean age of 61.7 (2.3). The most common etiological factors were biliary stones (in 61.6% of cases), followed by alcohol intake (26.6%) and hypertriglyceridemia (6.6%). 

### 3.2. Clinical Endpoints

**Figure 4:**
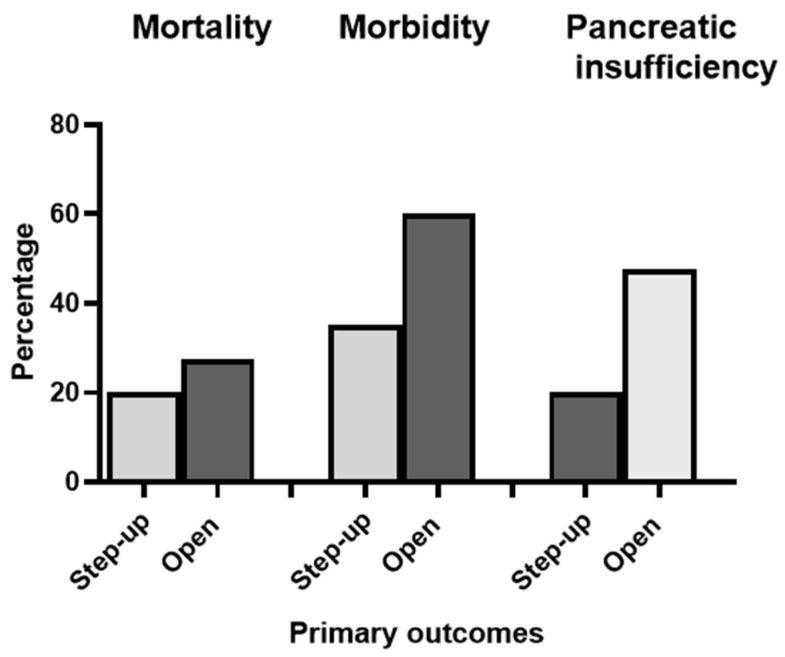
Main clinical outcomes.

**Figure 5:**
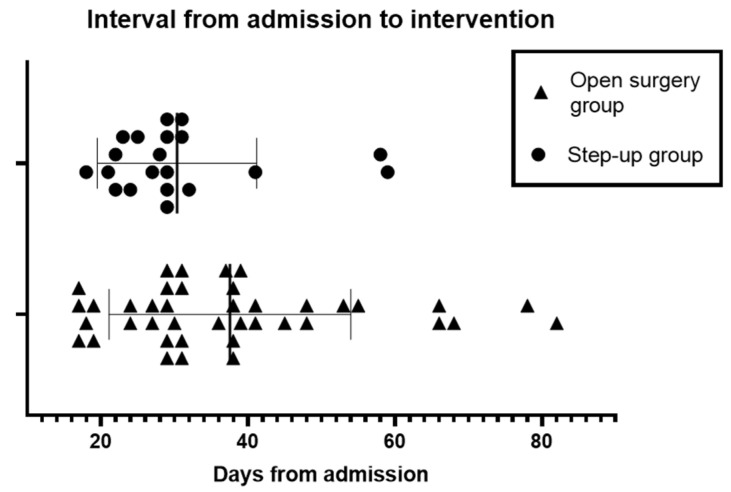
Graph showing the interval from admission to intervention for both groups.

Clinical endpoints are shown in Table 2 and Figure 4. A total of 15 (25%) patients in the study died within 6 months from the diagnosis. Mortality in the SUA group was 20% (4/20) and in ON group it was 27.5% (11/40); however, the mortality did not differ significantly (*p* = 0.52). The majority of patients died of multiple organ failure (three in the SUA group and eight in the ON group), two patients died of complications related to intraabdominal bleeding, and two had a respiratory failure due to pneumonia. Pancreatic necrosis was confirmed in 90% (54/60) of patients. The postoperative complication rate was 35% (*n* = 7) in the SUA group and 60% (*n* = 24) in the ON group (*p* = 0.04). Most frequently, reoperation was required for intra-abdominal sepsis (eight cases) and bleeding (two cases). Patients undergoing SUA had a shorter ICU stay (median of 11 versus 13 days) and hospital stay (median of 42 versus 51 days), although the difference was not statistically significant (*p* value of 0.37 for ICU stay and 0.44 for hospital stay). The mean number of necrosectomies (open or VARD) per patient was 0.95 in the SUA group and 1.6 in the ON group (mean difference: 0.65; 95% CI, 0.18 to 1.11). Time to intervention (TTI) is presented in Figure 5, which shows the distribution of TTI for every single patient. Most initial interventions were performed around the end of the 4th week of the disease. The ranges of initial intervention timing in the ON and SUA groups were 17–82 days and 18–59 days, respectively.

At the 1-year follow-up, patients who had undergone primary ON, as compared with patients who had been treated with the SUA, had a higher rate of pancreatic dysfunction (47.5% vs. 20%, *p* = 0.03). Endocrine insufficiency was more common than exocrine insufficiency in both groups.

### 3.3. Step-Up Approach Group

Nineteen patients in the step-up group underwent retroperitoneal percutaneous drainage while one patient underwent laparoscopic placement of a drain into the omental bursa. No patients underwent endoscopic (transgastric) drainage since it was not available at our institution during the study period. 

After the period of 72 h, a clinical improvement was observed in s patients (25%), and five of them recovered fully without the need for additional drainage or necrosectomy while another recovered after additional percutaneous drainage. More details about the procedure can be seen in Table 3. Overall, six patients required open necrosectomy eventually, and four of them died due to sepsis progression.

### 3.4. Open Necrosectomy Group

Median laparotomy was performed in 37 patients and bilateral transverse incisions in three patients. Sixteen patients underwent additional surgical necrosectomy (six of them more than two times). In a group of 24 patients who underwent a single necrosectomy, three of them died due to ongoing sepsis in the early postoperative period. Patients who required additional necrosectomy had a mortality rate of 50%. More specific data on outcomes and details about type interventions are presented in Table 2 and Table 3.

## 4. Discussion

Historically, ANP was associated with extremely high morbidity and mortality rates, and many clinical and scientific efforts have been made to improve outcomes following the most severe form of AP. There is still an ongoing debate regarding the benefit of medical or surgical treatment in ANP. Given the seriousness of ANP, some clinicians prefer surgical necrosectomy, even in the early phase [17,18]. Most, however, advocate initial conservative treatment and the reservation of surgery for abdominal catastrophes or ongoing sepsis related to infected pancreatic necrosis [7,8,9,10,11,12,13,14,15,16,19,20,21,22,23]. In the era of minimally invasive surgery and interventional radiology, consequently, new techniques and strategies to treat ANP have emerged. Clinicians hoped that this shift from early surgical debridement to a staged, minimally invasive step-up approach would result in a long-awaited substantial improvement in ANP outcomes. From the first report in 2010, which showed the superiority of SUA from both a clinical and an economic point of view, many studies confirmed a number of benefits of the step-up approach over laparotomy [2,3,5,6,16,18,19,20,23,24,25,26,27,28,29]. These include a lower rate of major complications, pancreatic fistulas, bleeding, hospital stays, and costs. Long-term follow-up showed comparable outcomes as well, and the PANTER trial also showed less organ failure, diabetes, and incisional hernia in the step-up group [2]. 

The rate of death between the groups was similar in the majority of studies; however, several authors reported better outcomes in terms of mortality for SUA [8,12,13,27]. It is worth noting that data were mostly derived from retrospective cohorts and single institutional reports, while only three were randomized, which suggests that further studies are required for high-quality evidence to be collected in order to define preferred therapy, optimal timing, and technical modifications of the SUA. Nevertheless, initial promising results have encouraged surgeons and gastroenterologists to adopt the minimally invasive approach in clinical practice and use it more frequently in the treatment of ANP with infected necrosis. In this study, we reported our experiences with patients undergoing surgical or minimally invasive necrosectomy for acute necrotizing pancreatitis with infected necrosis, including the effect of SUA on the endocrine and exocrine pancreatic functions, which had not been thoroughly studied by now.

Concerning the step-up approach, it is argued that it may be inefficient in infection source control and delay surgical treatment. Our study showed that only six patients from the step-up approach group required surgery eventually, and in each case, surgical intervention was not delayed. Moreover, there was no significant difference regarding time to intervention between the groups.

Another important finding is that no significant procedural complications of SUA were noticed, since there was a certain fear of iatrogenic injury to peripancreatic vessels and an inability to control bleeding. We recommend that debridement and lavage in the peripancreatic area using VARD be blunt but attentive and never too extensive.

Although a relatively small number of participants precludes drawing firm statistical conclusions related to mortality and survival rates, we demonstrated the non-inferiority of SU in terms of safety and efficiency. At the same time, it is associated with a lower complication rate, hospital/ICU stay, and costs. In addition, our results demonstrated that almost 30% (6/20) of patients in the SUA group were treated with percutaneous drainage only. These are patients that would otherwise undergo open necrosectomy, with all the risks that such a procedure carries.

Furthermore, we demonstrated a reduced rate of POF and pancreatic insufficiency at the 1-year follow-up. Pancreatic fistulas remained a significant problem in the postoperative period, with an overall rate of 36.6%, but most of these could have been treated by conservative or minimally invasive methods.

However, grade three fistulas were associated with the high mortality rate in both groups (66%). SUA had a lower rate of PF, but the difference was not statistically significant due to the low sample size. Nevertheless, the reduction in the PF rate may be an important advantage of SUA and the main cause of lower early postoperative morbidity when compared to ON.

The lower rate of pancreatic fistulas and pancreatic insufficiency in our study may be explained by less extensive surgical trauma when management is limited to minimally invasive methods only. Open necrosectomy has the disadvantage of generally long abdominal wall incisions and the opening of the peritoneum and omental bursa, which may be associated with bleeding, excessive necrosectomy, and the opening of routes for intra-abdominal abscess spreading.

On the other side, in the SUA approach, the intraperitoneal cavity is not opened, and necrosis and peripanceratic collections are drained only to suppress septic focus. This may be less effective in evacuating most necrotic masses than ON; however, as postulated by several authors, infected necrosis may be similar to an abscess under pressure, so simple drainage might be sufficient to treat it even if most of the pancreatic necrosis is left in situ [13,17,23]. The SUA requires radiological skills to perform the procedure, although it is relatively simple in institutions where other types of CT-guided drainages are mastered.

Similarly, VARD is a fairly straightforward procedure that can be performed by surgeons with basic laparoscopic skills and experience in the treatment of ANP. In contrast, the endoscopic step-up approach is more complex and requires the advanced skills of interventional gastroenterologists, although an RCT study by Brunschot et al. showed that the rate of pancreatic fistulas and length of hospital stay were lower in the endoscopy group when compared to the surgical step-up approach. However, in this study, the endoscopic step-up approach was not superior in reducing major complications or death [7].

Although the study was not designed as a controlled randomized trial (since the VARD method approach was not available in the early study period), meticulous case matching and prospective data collection were performed, which ensured that meaningful conclusions were drawn. The reliability of our results is supported by the fact that all main criteria of pancreatitis severity and patients’ clinical condition were comparable between the two groups (Ranson score, CT severity index, and Apache 2 score). Therefore, selection bias was maximally avoided, and clinically favorable patients were not more commonly assigned to the step-up approach group. We consider that future studies on ANP should focus on the optimal timing of intervention based on predictive factors, the definition of indications for SUA, and the timing of open surgery when SUA fails. Other questions that should be addressed are technical features such as the degree of VARD–necrosectomy, the route, and the size of percutaneous drains.

Finally, such studies on larger numbers of patients and randomized controlled trials may show us whether SUA is the preferred initial strategy for all patients with ANP without urgent surgical complications. This would leave open necrosectomy to be employed only after unsuccessful minimally invasive methods or in cases of critical clinical deterioration. Luckily, patients with ANP are, in most cases, already hospitalized for several weeks before infected necrosis develops, which allows sufficient time for the planning of optimal treatment. 

According to the published literature and our experience reported in this article, we consider that it should be the preferred method for infected pancreatic necrosis if there are technical and organizational capabilities [2,3,17,18,19,20,21,22,23,27,28,29]. The study was primarily designed to show if SUA may be employed in the same clinical scenarios as seen in the ON group of patients. Case matching according to disease severity allowed us to avoid selection bias and eliminate concerns that clinically better patients would be assigned to an SUA group. However, we think that SUA and ON should not be considered as alternatives to each other but rather as complementary methods in the APN treatment strategy. Such a variety of methods in the surgeon’s armamentarium may finally lead to considerable improvement in ANP outcomes.

## 5. Limitations

There are several limitations to the study. First, the study was not randomized since the step-up approach was introduced in the second half of the study period and not all surgeons were skilled to perform it. Thus, despite adequate case-matching, better results in the SUA group may be related to improved intensive care measures and better disease understanding in the latter part of the study period. Second, the unavailability of transgastric necrosectomy as originally described in the step-up approach precluded adequate comparison to previously published studies, so it would be interesting to see if endoscopic procedures could further reduce invasiveness in the future. Third, there was significant intragroup variability in Ranson score as shown by the wide confidence interval, indicating that the results should be interpreted with caution regardless of statistical significance. However, since both means and SDs were similar in both groups and no difference in CT severity score was observed, we may indirectly conclude that pancreatitis severity was comparable among the groups. Fourth, the sample size (especially in the SUA group) was too small for adequate analysis of statistical significance for primary outcomes, but due to the retrospective and single center design, we could not modify the number of participants nor randomize them. Therefore, it was not designed as non-inferiority study; nevertheless, the study results are informative and have clinical and scientific value in the field of minimally invasive pancreatitis treatment.

## 6. Conclusions

The management of infected necrosis in necrotizing pancreatitis has changed during the last decade due to the introduction of less invasive endoscopic and radiologic methods. A better understanding of the disease, selection of the optimal type and timing of intervention, and intensive care improvements have led to an improved prognosis in these patients. Our study demonstrated that the step-up approach may be a feasible strategy in the treatment of ANP, and it presents a significant contribution to the evaluation of SUA efficiency and safety. SUA may be another step forward in decreasing high mortality and morbidity in patients with APN and infected pancreatic necrosis.

## Figures and Tables

**Table 1:** Demographic and clinical characteristics.

Category	Step Up Approach	Open Necrosectomy	*p*-Value
Age	61.2 ± 2.3	62.4 ± 2.2	0.86
Male gender (*n*, %)	13 (65)	28 (70)	0.69
BMI, kg/m^2^	26 (23–28)	27 (23–29)	0.92
Ranson score, mean (±SD)	1.64 (±0.82)	1.67 (±0.91)	0.50
Etiology of ANP			
- Biliary	11 (55)	26 (65)	
- Alcohol-induced	6 (30)	10 (25)	0.71
- Other	3 (15)	4 (10)	
Apache II score	15.1 (±5.2)	15.9 (5.9)	0.71
CT severity index			
<50%	14	30	0.56
>50%	6	10	
Time from diagnosis to intervention, days (range)	29 (18–59)	32 (17–82)	0.08
Comorbidities			
- Cardiologic	9	26	0.59
- Pulmonary	3	5	0.32
- Renal	2	5	0.9
ASA score			
- I–II	13	28	0.77
- >II	7	2	
Serum lipase levels, U/l (± SD)	2456 (±2336)	2650 (±2211)	0.39
Leukocytes/mm^3^	18,096 (±9069)	19,244 (±8066)	0.44
Confirmed infected necrosis *n* (%)	18 (90)	36 (87.5)	1.0
Microbiological isolates			
*E. coli*	8	16	
*Klebsiella pneumoniae*	4	8	
*Acinetobacter* spp.	3	5	
*Pseudomonas aureginosa*	2	5	
*Enterococcum faecium*	1	3	
Other	2	3	

**Table 2:** Clinical endpoints.

Category	Step Up Approach	Open Necrosectomy	*p*-Value
**Mortality, *n* (%)**	4 (20)	11 (27.5)	0.53
Major morbidity, *n* (%)	7 (35)	24 (60)	0.044
▪ Single organ failure	3	8	
▪ Multiple organ dysfunction	1	4	
▪ Intra-abdominal bleeding	0	2	
▪ Intra-abdominal sepsis	3	6	
Pancreatic insufficiency, *n* (%)	4 (20)	19 (47.5)	0.038
▪ Endocrine	3	13	
▪ Exocrine	1	4	
▪ Both	0	2	
Hospital stay, median (range)	42 (9–212)	51 (22–255)	0.44
ICU stay, median (range)	11 (2–96)	13 (4–113)	0.37
Pancreatic fistula, *n* (%)	5 (25)	17 (42.5)	
▪ Grade I	2	8	0.18
▪ Grade II	1	5	
▪ Grade III	2	4	
Number of interventions per group			
▪ Including percutaneous drainage	32	96	0.04
▪ Excluding percutaneous drainage	21	79	

**Table 3:** Procedural characteristics.

**Group**	**Percutaneous Drainage Only**		**Percutaneous Drainage + VARD Only**		**Percutaneous Drainage + VARD + Open Necrosectomy**		**Percutaneous Drainage + Open Necrosectomy**	
	Single	Multiple	Single VARD	Multiple VARDs	Single necrosectomy	Multiple necrosectomies	Single	Multiple
Step-up approach	5	1	6	2	3	2	1	0
**Group**	**Single Open Necrosectomy**		**Multiple Open Necrosectomies**		**Additional** **Percutaneous Drainage**		**Additional Surgery for Complications**	
			2	>2	Single	Multiple	Single	Multiple
Open necrosectomy	24		10	6	8	6	5	3

## Data Availability

Data is unavailable due to privacy or ethical restrictions.

## References

[1] Leonard-Murali S., Lezotte J., Kalu R., Blyden D.J., Patton J.H., Johnson J.L., Gupta A.H. (2021). Necrotizing Pancreatitis: A Review for the Acute Care Surgeon. Am. J. Surg..

[2] Van Santvoort H.C., Besselink M.G., Bakker O.J., Hofker H.S., Boermeester M.A., Dejong C.H., Van Goor H., Schaapherder A.F., Van Eijck C.H., Bollen T.L. (2010). A Step-up Approach or Open Necrosectomy for Necrotizing Pancreatitis. N. Engl. J. Med..

[3] Miskovitz P. (2015). A Step-Up Approach to Managing Acute Pancreatitis–Associated Fluid Collections. Crit. Care Med..

[4] Baron T.H., DiMaio C.J., Wang A.Y., Morgan K.A. (2020). American Gastroenterological Association Clinical Practice Update: Management of Pancreatic Necrosis. Gastroenterology.

[5] Bang J.Y., Arnoletti J.P., Holt B.A., Sutton B., Hasan M.K., Navaneethan U., Feranec N., Wilcox C.M., Tharian B., Hawes R.H. (2019). An Endoscopic Transluminal Approach, Compared with Minimally Invasive Surgery, Reduces Complications and Costs for Patients with Necrotizing Pancreatitis. Gastroenterology.

[6] Jones J.D., Clark C.J., Dyer R., Case L.D., Mishra G., Pawa R. (2018). Analysis of a Step-Up Approach Versus Primary Open Surgical Necrosectomy in the Management of Necrotizing Pancreatitis. Pancreas.

[7] Van Brunschot S., Van Grinsven J., Van Santvoort H.C., Bakker O.J., Besselink M.G., Boermeester M.A., Bollen T.L., Bosscha K., Bouwense S.A., Bruno M.J. (2018). Endoscopic or Surgical Step-up Approach for Infected Necrotising Pancreatitis: A Multicentre Randomised Trial. Lancet.

[8] Wundsam H.V., Spaun G.O., Bräuer F., Schwaiger C., Fischer I., Függer R. (2019). Evolution of Transluminal Necrosectomy for Acute Pancreatitis to Stent in Stent Therapy: Step-up Approach Leads to Low Mortality and Morbidity Rates in 302 Consecutive Cases of Acute Pancreatitis. J. Laparoendosc. Adv. Surg. Tech. Part A.

[9] Boxhoorn L., Van Dijk S.M., Van Grinsven J., Verdonk R.C., Boermeester M.A., Bollen T.L., Bouwense S.A.W., Bruno M.J., Cappendijk V.C., Dejong C.H.C. (2021). Immediate versus Postponed Intervention for Infected Necrotizing Pancreatitis. N. Engl. J. Med..

[10] Luckhurst C.M., Hechi M.E., Elsharkawy A.E., Eid A.I., Maurer L.R., Kaafarani H.M., Thabet A., Forcione D.G., Castillo C.F.-D., Lillemoe K.D. (2020). Improved Mortality in Necrotizing Pancreatitis with a Multidisciplinary Minimally Invasive Step-Up Approach: Comparison with a Modern Open Necrosectomy Cohort. J. Am. Coll. Surg..

[11] Liu Z., Yang S., Wang P., Feng J., He L., Du J., Xiao Y., Jiao H., Zhou F., Song Q. (2020). Minimal-Access Retroperitoneal Pancreatic Necrosectomy for Infected Necrotizing Pancreatitis: A Multicentre Study of a Step-up Approach. Br. J. Surg..

[12] Sion M.K., Davis K.A. (2019). Step-up Approach for the Management of Pancreatic Necrosis: A Review of the Literature. Trauma Surg. Acute Care Open.

[13] Besselink M.G.H., Van Santvoort H.C., Nieuwenhuijs V.B., Boermeester M.A., Bollen T.L., Buskens E., Dejong C.H.C., Van Eijck C.H.J., Van Goor H., Hofker S.S. (2006). Minimally Invasive “step-up Approach” versus Maximal Necrosectomy in Patients with Acute Necrotising Pancreatitis (PANTER Trial): Design and Rationale of a Randomised Controlled Multicenter Trial [ISRCTN13975868]. BMC Surg..

[14] Dindo D., Demartines N., Clavien P.-A. (2004). Classification of Surgical Complications. Ann. Surg..

[15] Bassi C., Dervenis C., Butturini G., Fingerhut A., Yeo C., Izbicki J., Neoptolemos J., Sarr M., Traverso W., Buchler M. (2005). Postoperative Pancreatic Fistula: An International Study Group (ISGPF) Definition. Surgery.

[16] Gasparoto R.C.G.W., De Castro Jorge Racy M., De Campos T. (2015). Long-Term Outcomes after Acute Necrotizing Pancreatitis: What Happens to the Pancreas and to the Patient?. J. Pancreas.

[17] Trikudanathan G., Wolbrink D.R.J., Van Santvoort H.C., Mallery S., Freeman M., Besselink M.G. (2019). Current Concepts in Severe Acute and Necrotizing Pancreatitis: An Evidence-Based Approach. Gastroenterology.

[18] Kempeneers M.A., Besselink M.G., Issa Y., Van Hooft J.E., Van Goor H., Bruno M.J., Van Santvoort H.C., Boermeester M.A. (2017). Multidisciplinary Treatment of Chronic Pancreatitis: An Overview of Current Step-up Approach and New Options. Ned. Tijdschr. Voor Geneeskd..

[19] Jain S., Padhan R., Bopanna S., Jain S.K., Dhingra R., Dash N.R., Madhusudan K.S., Gamanagatti S.R., Sahni P., Garg P.K. (2019). Percutaneous Endoscopic Step-Up Therapy Is an Effective Minimally Invasive Approach for Infected Necrotizing Pancreatitis. Dig. Dis. Sci..

[20] Sundaramurthi S., Kannan A., Nagarajan R., Dasarathan S., Dharanipragada K. (2021). Precise Pancreatic Necrosectomy by Step-up Approach. EXCLI J..

[21] Maurer L.R., Maatman T.K., Luckhurst C.M., Horvath K.D., Zyromski N.J., Fagenholz P.J. (2021). Risk of Gallstone-Related Complications in Necrotizing Pancreatitis Patients Treated with a Step-up Approach: The Experience of Two Tertiary Care Centers. Surgery.

[22] Gomes C., Di Saverio S., Sartelli M., Segallini E., Cilloni N., Pezzilli R., Pagano N., Gomes F., Catena F. (2020). Severe Acute Pancreatitis: Eight Fundamental Steps Revised according to the ‘PANCREAS’ Acronym. Ann. R. Coll. Surg. Engl..

[23] Da Costa D.W., Boerma D., Van Santvoort H.C., Horvath K.D., Werner J., Carter C.R., Bollen T.L., Gooszen H.G., Besselink M.G., Bakker O.J. (2013). Staged Multidisciplinary Step-up Management for Necrotizing Pancreatitis. Br. J. Surg..

[24] Zheng Z., Lu J.-D., Ding Y.-X., Guo Y.-L., Mei W.-T., Qu Y.-X., Cao F., Li F. (2021). Comparison of Safety, Efficacy, and Long-Term Follow-up between “One-Step” and “Step-up” Approaches for Infected Pancreatic Necrosis. World J. Gastrointest. Surg..

[25] Morató O., Poves I., Ilzarbe L., Radosevic A., Vázquez-Sánchez A., Sánchez-Parrilla J., Burdío F., Grande L. (2018). Minimally Invasive Surgery in the Era of Step-up Approach for Treatment of Severe Acute Pancreatitis. Int. J. Surg..

[26] Aparna D., Kumar S., Kamalkumar S. (2017). Mortality and Morbidity in Necrotizing Pancreatitis Managed on Principles of Step-up Approach: 7 Years Experience from a Single Surgical Unit. World J. Gastrointest. Surg..

[27] Cao F., Duan N., Gao C., Li A., Li F. (2019). One-Step Verse Step-Up Laparoscopic-Assisted Necrosectomy for Infected Pancreatic Necrosis. Dig. Surg..

[28] Huang D., Lu Z., Li Q., Jiang K., Wu J., Gao W., Miao Y. (2023). A Risk Score for Predicting the Necessity of Surgical Necrosectomy in the Treatment of Infected Necrotizing Pancreatitis. J. Gastrointest. Surg..

[29] Delgado S.C., Martinez A.V., Sánchez T.G., Aguilar A.F., García J.M.P. (2022). Step-up Approach in Severe Necrotizing Pancreatitis: Combination of Video-Assisted Retroperitoneal Debridement and Endoscopic Necrosectomy. Cirugía Española.

